# Why do tumor-infiltrating lymphocytes have variable efficacy in the treatment of solid tumors?

**DOI:** 10.3389/fimmu.2022.973881

**Published:** 2022-10-21

**Authors:** Biaoru Li

**Affiliations:** Georgia Cancer Center and Department of Pediatrics, Medical College at Georgia (GA), Augusta, GA, United States

**Keywords:** TILs (tumor infiltrating lymphocytes), solid tumor, TIL quiescence, TIL attacking heterogeneous antigen, TIL infiltration, personalized immunotherapy

## Abstract

Lymphocytes in tumor tissue are called tumor-infiltrating lymphocytes (TILs), and they play a key role in the control and treatment of tumor diseases. Since the discovery in 1987 that cultured TILs can kill tumor cells more than 100 times more effectively than T-cells cultured from peripheral blood in melanoma, it has been confirmed that cultured TILs can successfully cure clinical patients with melanoma. Since 1989, after we investigated TIL isolation performance from solid tumors, we modified some procedures to increase efficacy, and thus successfully established new TIL isolation and culture methods in 1994. Moreover, our laboratory and clinicians using our cultured TILs have published more than 30 papers. To improve the efficacy of TILs, we have been carrying out studies of TIL efficacy to treat solid tumor diseases for approximately 30 years. The three main questions of TIL study have been “How do TILs remain silent in solid tumor tissue?”, “How do TILs attack homologous and heterologous antigens from tumor cells of solid tumors?”, and “How do TILs infiltrate solid tumor tissue from a distance into tumor sites to kill tumor cells?”. Research on these three issues has increasingly answered these questions. In this review I summarize the main issues surrounding TILs in treating solid tumors. This review aims to study the killing function of TILs from solid tumor tissues, thereby ultimately introducing the optimal strategy for patients suffering from solid tumors through personalized immunotherapy in the near future.

## Introduction

Tumor-infiltrating lymphocyte (TIL) therapy, one of the best adoptive immunotherapies (AITs) or adoptive cell therapies (ACTs), was discovered about 35 years ago, when Dr Steven Rosenberg used TILs to treat melanoma in 1988 (1–3). At a similar time, we were studying TIL cytotoxicity and the killing of tumor cells from solid tumors (4–10). After studying TIL cytotoxicity primarily related to T-cell isolation from solid tumors, we modified procedures and successfully used TIL isolation from solid tumors to treat several hundred patients suffering from solid tumors. We have produced more than 30 related publications since 1994 (11–18).

Although TIL immunotherapy has been broadly accepted to treat melanoma, variable responses mean that the efficacy of TILS in treating solid tumor diseases is still questioned (19, 20). Despite the fact that TILs are increasingly seen to have benefits that make them applicable for cancer treatment in some clinical laboratories, two conflicting opinions on efficacy still remain with regard to TIL immunotherapy to treat solid tumors. The first is that, even if TIL responses are powerful, undergoing a 1,000-fold expansion after activation (1), some reports show that TIL immunotherapy could not reliably produce effective responses in treating solid tumors in other clinical laboratories, especially in some early research reports (21, 22). The second issue is variable results to assay the cytotoxicity of tumor-specific lymphocytes to tumor cells for different tumor diseases (23). According to several laboratories’ reports, the anti-tumor cytotoxic activity of TILs can be identified in tumor samples of more than 80% of patients with melanoma, whereas the results from solid tumor diseases fluctuate (24, 25). However, two reports suggest that TILs have promising prospects; for example, they report TILs specifically traveling into the site of a solid tumor mass to kill tumor cells and TILs maintaining their therapeutic efficacy for many years after initial treatment (26, 27).

Because TILs actively killing human tumor cells from solid tumors remains controversial, and to address the auguring, this review presents three issues regarding the efficacy of TILs from solid tumors. Finally, I briefly present the future of adoptive TIL immunotherapy as strategies of personalized immunotherapy (28).

## Why do TILs have variable efficacy in the treatmeant of solid tumors?

After Dr Rosenberg discovered that TILs can be cultured with the aid of the cytokine interleukin (IL)-2 and induced TIL-exhibited cytotoxic activity against melanoma cells *in vitro*, TILs isolated from tumor samples were studied in the earliest trials of ACT conducted at the surgical branch of the National Cancer Institute (NCI) in 1988 (29, 30). At that time, objective responses were observed in 11 out of 20 patients with metastatic melanoma. Five out of the 29 (17%) responses were complete responses (CRs), with a median duration of response of 4 months in these early studies (31). After more than 30 years of research and development (R&D), TILs have shown promising overall response rates (38%) for the treatment of recurrent/refractory melanoma according to clinical trial results presented at the 2021 American Society of Clinical Oncology (ASCO) Annual Meeting (32). Now, patients are first treated with cyclophosphamide (or fludarabine) (lymph depletion) for 1 week, followed by TIL infusion and then six doses of adjuvant IL-2. The TIL manufacturing process takes about 22 days (33).

Through studying TIL cytotoxicity mechanisms to autologous tumor cells from solid tumors during that early period, we discovered that TILs had variable efficacy in the treatment of patients with solid tumors (6, 34). To address how to increase TIL efficacy in the treatment of solid tumors, we began to study all procedures regarding TIL purification and culture from solid tumors. After spending approximately 30 years studying this, here I summarize the three issues discovered through the investigations as Silencing TILs in the tumor location, Heterogeneous immune response from heterogeneous tumor antigens, and Infiltrating TIL into solid tumors. I refer to these three issues as the “SHI” phenomenon (“SHI” in Chinese translates as “STONE”, whereas in English it is the same as the traditional Chinese medicine term for “tumor disease”).

### Silencing TILs

In 1995, after analyzing TIL proliferation, cytotoxicity, and phenotype from 83 solid tumor cases, we reported that TILs have quiescent status if the TIL is located in solid tumor tissues (35). Although we discovered freshly isolated TIL-containing activated T-cell surface markers such as Human Leukocyte Antigen – DR isotype (HLA DR) and IL-2 receptors, the cells did not have strong cytotoxicity against tumor cells. In this early study, results regarding silencing TILs showed that the supernatants of TIL culture media contained inhibiting factors, including some things from autologous tumor cells. To overcome the inhibiting factors, we studied them and modified our methods, such as eliminating autologous tumor cells during TIL culture procedures, so that in this early period we achieved good responses to increasing cytotoxicity to autologous tumor cells from solid tumors (36). After a long period of study, now clinical scientists and clinical immunologists understand that there are two major factors in TIL quiescence influencing efficacy: internal factors and external factors.

#### Internal mechanism

Silencing T-lymphocytes (quiescent lymphocytes) are a group of T-cells that display no spontaneous proliferation and a low metabolic rate (37). In silencing lymphocytes, quiescence reduces consumption of resources to maintain the vast repertoire of lymphocytes. Only a small fraction of native lymphocytes will be clonally selected by antigens during the lifetime of the host. From observation of lymphocyte quiescence, six mechanisms have been proposed to produce quiescence of lymphocytes (38–42): (1) thymus negative selection, (2) peripheral clonal deletion, (3) peripheral-induced anergy, (4) T-cell ignorance/indifference, (5) T-cell suppression, and (6) T-cell senescence (exhaustion). Lymphocyte quiescence obtained from solid tumors is caused by one or more factors of the six mechanisms, and, therefore, TIL quiescence may or may not produce immune cytotoxicity to tumor cells. To elucidate TIL quiescence from solid tumors, we screened and harvested quiescent TILs from those of hepatic cell cancers (HCCs). Before the genomic era (1996–2004), we used a single-cell messenger ribonucleic acid (mRNA) display system to find a profile from the quiescent cluster of differentiation 8 (CD8) TILs (43–45). After that, because microarray and ribonucleic acid sequencing (RNA-seq) began to be applied for single-cell genomic analyses, we carried out further studies of the set of quiescent genes by single-cell RNA-seq, as mentioned below. Now, most of the TIL quiescent genes have been confirmed by using single-cell quantitative reverse transcription polymerase chain reaction (RT-PCR) from both single-cell mRNA displays and single-cell RNA-seqs. The genes comprised at least seven genes, including Tob, LKLF (lung Krüppel-like factor), transforming growth factor (TGF)-beta, Sno, Ski, RE1 silencing transcriptor (REST) and ETS2 repressor factor (ERF). Nowadays, several laboratories have increasingly supported the results; as shown in **Table 1**, more genes, such as BTB Domain and CNC Homolog 2 (BACH2), Forkhead Box O1 (FOXO1) and Signal Transducer and Activator Of Transcription 3 (STAT3), have been found to be involved in quiescent lymphocytes. This finding comes after more than two decades of studying.

**Table 1:** Higher expression genes from quiescent T-cells.

Gene	Discovery of quiescent T-cell	Discovery of quiescent TIL		
	Source/year/journal	Source	Publication year	Journal
**Tob**	Quiescent T-cells/2001/Nat Immunology	Hepatic Ca	2007	Immunology
**KLF2**	Quiescent T-cells/2001/Nat Immunology	Hepatic/Colorectal cancer	2007/2019	Immunology/Cancer Immunology Research
**Ski**	Oncoimmune Response/2021/Front Immunology	Hepatic Ca	2007	Immunology
**Sno-A**	Mice Model/2003/MCB	Hepatic Ca	2007	Immunology
**TGF-beta**	TGF-beta pathway/1991/JEM	Hepatic Ca	2007	Immunology
**ERF**	Quiescent T-cells/1990/PNAS	Hepatic Ca	2007	Immunology
**REST**	Quiescent T-cells/2018/Mol Science	Hepatic Ca	2007	Immunology
**TCF-1**	Quiescent T-cells/2018/Mol Science	Ovarian cancer/Pancreatic Ca	2019	J. Immunology Cancer
**Bach-2**	Quiescent T-cells/2018/Mol Science	GC	2021	Aging
**FOXO1**	Quiescent T-cells/2011/JBC	Lung Ca	2018	Cancer
**STAT3**	Quiescent T-cells/2011/JBC	N/Y	N/Y	N/Y

N/Y is "unclear".

As shown in **Table 1**, the data for which come from different research laboratories, TOB (transducer of ERBB2) is a negative regulator of IL-2 transcription and T-cell for T-cell proliferation, which inhibits the Ag-MHCII pathway (46). LKLF (or KLF2) is a zinc finger-containing transcription factor that plays a negative regulatory role in cytotoxic T-lymphocyte (CTL), killing tumor cells by blocking the mimicry of IL2, tumor necrosis factor (TNF)-α, and interferon (IFN)-γ (47). Ski, Sno and TGF-β are involved in the TGF-β pathway to maintain T-cell quiescence (48, 49). ERF negatively regulates TIL infiltration, migration, and migration to tumor sites (50). The REST gene, like PD-1 and CTLA-4, is involved in blocking the PI3K pathway (51). There are increasing reports of The transcription factor T cell factor 1 (TCF-1), Bach-2, FOXO1 and STAT3 as Krüppel-like Factor 2 (KLF2), which can inhibit T-cells blocking the Cytotoxic T lymphocytes (CTL) pathway (52).

#### External factors

As mentioned above, in 1994 we reported that TIL proliferation and cytotoxicity during TIL culture were inhibited by autologous tumor cells from solid tumors (35). To increase the expansion and cytotoxicity of TILs, we performed clean procedures during the isolation and culture of TILs, such as the adhesion process that clears autologous tumor cells from HCC and lung cancer (36). After two decades of effort, it was discovered that the so-called tumor microenvironment (TME) suppression phenomenon can maintain TIL quiescence and affect TIL cytotoxicity during TIL culture (53). The TME consists of three components (54–60): (1) a tissue called the extracellular matrix (ECM) with epithelium, basal and endothelium; (2) regulatory cells including tumor-associated macrophages (TAMs), neutrophils (tumor-related neutrophils-2, TAN-2), cancer-associated fibroblasts (CAFs), and myeloid-derived suppressor cells (MDSCs); and (3) signaling molecules by releasing extracellular signals, promoting tumor angiogenesis, promoting tumor cell growth and affecting tumor growth. Lymphocyte quiescence of the TME has been extensively reported *in vivo*, *in vitro*, and *ex vivo*. All ECMs have been shown to produce TGF-β, which blocks TIL function, including the inhibition of TIL growth and cytotoxicity (61). In addition, some signaling molecules can affect the growth of TILs by releasing extracellular signal pathway, such as adenosine (ADO) signaling molecules and indole 2,3-dioxygenase (IDO) regulatory molecules (62, 63). Adenosine triphosphate (ATP) is a popular molecule that plays a vital role as a universal energy currency within cells. ATP induces immunogenic cell death (ICD) of tumor cells at the tumor site and promotes immune surveillance in the TME, whereas ADO increases the lead to ADO immune dysfunction in T-cells, NK cells, and B cells at the tumor site. A secondary regulatory pathway that impedes T-cell proliferation in the TME is the IDO pathway. Dendritic cells (DCs,) myeloid-derived suppressor cells (MDSCs) and tumor cells can produce IDO, which breaks down tryptophan and produces kynurenine, resulting in tryptophan deprivation and production of its metabolites to inhibit the expansion of clonal T-cells.

### Heterogeneous immune response from heterogeneous tumor antigens

To learn more about the immune response of TILs to autologous tumor cells, we have spent about 15 years studying the different TIL responses with their genes from solid tumors through single-cell gene expressions and individual immune responses in networks. After these studies from our experiments or from other data, we learned that TILs will produce responses to autologous tumor cells, including self-tolerance to homologous antigens, heterogeneous responses, and heterogeneous networks from TILs to autologous tumor cells.

#### Self-tolerance against homologous antigens

Heterogeneous immune responses and homologous immune responses are some of the key issues in addressing the efficacy of TILs on autologous tumor cells (64). Effective TIL CD8 cytotoxicity against autologous tumor cells results in TIL efficacy (65). Human tumor antigens are divided into two main types: shared tumor-associated antigens (TAAs) and tumor-specific antigens (TSAs). TAAs include (1) cancer-testis (CT) antigens, (2) differentiation antigens (DAs) and (3) onco-fetal antigens (OFAs). TSAs include neo-antigens and tumor viral antigens such as hepatitis B (HBV), Epstein-Barr Virus (EBV), cytomegalovirus (CMV), and human papillomavirus (HPV) E6/E7 proteins.

CT antigens (such as MAGE-1) are present for some time during the spermatogenesis and placental stages, and they are also more highly expressed in different types of neoplastic diseases (66). Normally they are silent in adult tissues, and, therefore, the transcriptional machinery is stimulated in certain tumor types. Although many types of tumors express CT antigens at high levels and corresponding normal tissues at low levels, they are sometimes expressed at high levels in normal tissues. Differentiation antigens (such as gp100) are encoded by genes that are expressed in a tissue-specific manner (67). These proteins are usually produced in very low amounts, but their production is dramatically increased in tumor cells to activate immune responses. An example of such a protein is tyrosine, which is required for melanin production. They also share antigens between tumor cells and corresponding normal cells. Most of these antigens, such as gp100 glycoprotein and MART-1, are mainly present in metastatic melanoma in solid tissues (68, 69). Carcinoembryonic antigens are the third important TAA tumor antigens, such as alpha fetoprotein (AFP) and carcinoembryonic antigen (CEA) (70, 71). AFP is highly expressed in hepatocellular carcinoma (HCC) the most common type of primary liver cancer , and CEA is often highly expressed in colon cancer and other tumors such as NSCLC (non-small cell lung cancer). CT antigen, differentiation antigen, and onco-fetal antigen are produced early in the embryonic development stage and disappear when the adult immune system is fully developed, so self-tolerance may result in quiescent TILs against these antigens (72).

#### Heterogeneity from T-cell neo-antigens

Neo-antigens caused by genetic instability during carcinogenesis appear in non-coding and coding regions, whereas amino acid sequence changes caused by mutations in coding regions can generate antigens that do not exist in normal cells (73). Neo-antigens can also be induced by viral infection, such as alternative splicing and gene rearrangements (74). These variant antigens can be recognized by immune cells, such as TILs, and are, theoretically, specific immune responses to tumor cells, without autoimmunity to autologous normal cells.

There are two types of neo-antigens: shared neo-antigens and personalized neo-antigens (75, 76). Shared neo-antigens refer to mutated antigens that are common in different cancer patients and not present in the normal genome, have a high immunogenic potential for screening, and are now used as broad-spectrum therapeutic cancer vaccines or adoptive immunotherapy for cancer patients, as shown in **Table 2**. Personalized neo-antigens refer to mutated antigens that differ from patient to patient (77, 78). Therefore, personalized neo-antigens can be targeted specifically for each patient, which can be used to design personalized immunotherapy.

**Table 2:** Neo-antigen of common solid tumors (TCGA).

Gene	AA change	Pancreas	Colorectal	ESCC	Liver	Lung adenocarcinoma	Lung squamous cell carcinoma	Ovarian	Stomach	Cervical
		(QCMG 2016)	(TCGA)	(UCLA 2014)	(TCGA)	(TCGA)	(TCGA)	(TCGA)	(TCGA)	(TCGA)
TP53	R175H	**3.9%**	**6.3%**	0.7%	–	**1.3%**	**–**	**2%**	**2.8%**	**–**
	R173H	**2%**	**3.1%**		**–**	0.4%	0.6%	**3.2%**	**2.3%**	**–**
	R273C	**2.1%**	**2%**	0.7%	–	0.4%	**1%**	**2.2%**	**2.5%**	**–**
	R248W	**1%**	**3.6%**	0.7%	0.3%		**1%**	**1.6%**	**1%**	
	R2480	**1.8%**	0.4%	–	**1%**	**–**	**0.6%**	**2.5%**	**1.5%**	
	R282W	**3%**	**1%**			0,4%	**1%**	**1.6%**	**1.8%**	
	Y220C	**1%**		0.7%	**1%**	0.4%	**1.1%**	**3%**	**1%**	
	V157F	0.8%		**1.5%**	**1%**	0.4%	**1.7%**	**1.6%**		
	G2455	**1.6%**	**2%**		**–**	**–**	**1%**	**0.9%**	0.5%	–
	Y163C	0.8%		**1.5%**		**–**	**2%**	**1%**	**–**	**–**
	R2495	0.5%	–	–	**3%**	**–**	**0.6%**	**–**	**1%**	**–**
KRAS	012D	**35.5%**	**13.9%**		0.5%	**2.2%**			**2.8%**	**2.1%**
	G12V	**27.9%**	**10.3%**	**–**	**–**	**9%**	**–**	0.6%	0.8%	**1%**
	G12C	**1.6%**	**3%**		0.3%	**15.7%**		**–**	0.3%	**1%**
	G12R	**15.7%**	0.4%	–	–	–	–	–	–	–
	G130	0.3%	**4.5%**						**3%**	**1%**
	**Q61H**	**5%**					**0.6%**		**0.5%**	
	012A	0.5%	1%			**2.6%**				
	012S		**1.3%**		**–**	**1%**			**1%**	
PIK3CA	E542K	0.3%	0.9%	0.7%	0.3%	**1.3%**	**1.7%**	**–**	**1.8%**	**6.2%**
	**E545K**	0.3%	**3.6%**	**2.9%**	0.3%	**2.2%**	**5.6%**	0.3%	**3%**	**12.9%**
	H1047R	0.5%	**2.2%**	**1.5%**	0.8%	0.4%	**1.1%**	0.3%	**3.3%**	0.5%
CTNNB1	S45P	–	–	–	**3%**	**–**	**–**	**–**	**–**	**–**
	T41A				**1.6%**	0.4%				
EGFR	L858R	–	–	–	–	**3.5%**	**–**	**–**	**–**	**–**
	T790M			–		0.4%	–	–	–	–
BRAF	**V600E**	**–**	**9%**	**–**		**2.2%**	**–**	**–**	**–**	**–**
GNAS	R201C	1%			1.1^0^/0				0.3%	
	R201H	0.8%	0.4%			0.9%			0.8%	**0.5%**

Incidence of mutated antigens in each tumor.

Different types of neo-antigens (TSAs) and different amounts of TAAs can exist in the same tumor in different individuals, resulting in individual immune responses, so TIL administration should be developed into an individualized design for immunotherapy. Based on personalized information such as individual genomic data, personalized TIL therapy can increase the intensity and durability of antitumor effects, improve survival and quality of life, and ultimately improve cancer treatment outcomes for patients. For most patients with solid tumors, personalized TIL therapy is expected to be more feasible and safe.

#### Heterogeneous network response

To investigate individualized immune responses in different patients, we have studied TIL responses with distinct networks, even if their TILs were derived from patients with nearly similar clinical and pathological outcomes. Under analysis of their pathways and networks, through achieving gene expression patterns by single-cell gene expressions and analyzing molecular expressions from the heterogeneous responses of TIL CD8 cells, we found that small gene expression changes between two TILs can reveal individual immune responses in the network. A network study (79) of two TIL CD8 cells from two patients showed that Tob-1 gained some similar higher expressions, suggesting a common immune response in its quiescent network. Once one or more proteins are added to the network, changes in the network can occur. For example, TGFB1 and ERF underwent a greater increase in sample 1, whereas Sno-A and REST underwent a greater expression in sample 2, so, eventually, the granzyme B and perforin in CTL expressed were completely different. The results demonstrate that TILs can reveal individual immune responses in the network, and thus, artificial intelligence network analysis can be used in TIL personalized immunotherapy.

### Infiltrating TILs into tumor tissue to attack tumor cells

The infiltration of TILs into tumor tissue to kill tumor cells has been studied in two areas (1): TIL CD8 cells should have intact signaling molecules after harvesting TILs from tumor tissue (4–6, 80), and (2) how *ex vivo*-cultured TILs specifically enter tumor tissue from circulating blood after reinfusion (81). The first question has been studied by our colleagues for more than 30 years.

#### CTL signal intactness

Earlier, we found changeable results based on Dr Rosenberg’s tumor disaggregation (with triple enzymes by collagenase type-IV, hyaluronidase, and DNase) to harvest and culture TILs for clinical treatment if we used the disaggregation procedures from solid tumors. Considering the TIL results in experimental and clinical work to treat solid tumors, we studied the TIL functions such as TIL proliferation, cytotoxicity, and phenotype with three enzymes under their conditions (37°C) (5). Finally, after modifying the enzymes’ condition, once collagenase IV under very moderate digestion condition the TILs’ function can be kept with an optimal proliferation, activity, and cytotoxicity to kill autogenous tumor cells. As stated in earlier publications (5, 6, 82), we had isolated and cultured TILs from solid tumors using the mild enzymatic digestion (cold enzymatic digestion with collagenase IV only), and the results showed that 65% of TILs proliferated more than 1,000 fold. The (3)TdR incorporation rate peaked at 45–75 days. Cytotoxicity to tumor cells was maintained for 56 days. The phenotypes of TILs after IL-2 induction were CD3 80 ± 21%, CD4 37 ± 21%, CD8 44 ± 18%, and HLA DR 69 ± 24%. CD3 and CD8 were significantly higher than in other clinical laboratories. Our clinical trials to treat solid tumors had shown better treatment outcomes than data from other clinical laboratories (7). Since then, most clinical laboratories chose collagenase IV only to disaggregate tumor tissues to harvest TILs. After 30 years’ study, we discovered that current collagenase IV products still contain other trace protease functions, such as trypsin-like activity. Collagenase IV products influence intact molecules on CD8 cells because abundant molecules are kept on the surface of TIL CD8 cells. Now, we are going to study the collagenase structure to influence TIL functions on CD8 cells. Hopefully, the functionally or genetically modified collagenase IV will delete trypsin-like activity. All in all, even if the trypsin activity of collagenase products is very low, abundant molecules on the surface of TIL CD8 cells, which are influenced by collagenase, are important for the adoptive TIL immunotherapy to solid tumors (4, 83).

##### TIL moving, migration, and infiltrating

Regardless of whether TILs can enter tumor tissue after isolation, culture, and infusion of TILs for immunotherapy, in early events, TILs cannot be fully characterized as tumor-specific T-cells *in vivo*. The mobilization of TILs into tumor tissue has become increasingly accepted after two experiments: Dr Torcellan’s *in vivo* TIL light-labeling technique in 2017 and Bai’s use of on-site antigen presentation for clonal expansion in 2001 (84, 85).

Despite the fact that T-cells play an important role at the tumor site, the specific transport capacity of TILs is dependent on dynamic processes such as rolling, adhesion, extravasation, and chemotaxis, as shown in **Table 3** (86, 87). Because successfully killing tumor cells in solid tumors relies on a high frequency of TILs, TILs that target tumor cells may also include TIL homing processes influenced by immunosuppression and abnormal vasculature (88, 89).

**Table 3:** TIL infiltrating genes.

Genes	Function	Correspondent partner
**Selectin ligand**	Rolling	Vascular E/P selectin
**VLA-4**	Adhesion	Vascular VCAM-4
**LFA-1**	Adhesion	Vascular ICAM-1
**TNF-alpha**	Extravasation	Vascular epithelium
**IL-6**	Extravasation	Vascular epithelium
**CXCR 1/2**	Chemotaxis	Tumor location (CCL)
**CXCR 3/4**	Chemotaxis	Tumor location (CXCL)

Now, CXCR2 (the receptor for CXCL1 secreted by tumor cells), CCR4 (CCL17, the ligand for CCR4), and others have been modified to enhance T-cell trafficking to the tumor site (90–92). These studies suggest that future combination therapy may enhance the efficacy and homing ability of adoptively transferred T-cells in patients.

## How can TILs have stable efficacy in the treatment of solid tumors?

**Figure 1:**
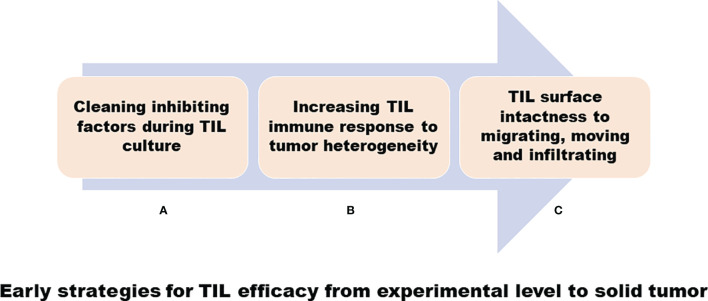
Early strategies for TIL efficacy from experimental level to solid tumor. **(A)** A cleaning inhibiting factor such as removing tumor cells during TIL cultures. **(B)** Increasing TIL immune response to tumor cells, including transducing TNA-α gene into TIL for heterogeneous tumor antigens. **(C)** Mild collagenase IV digestion to disaggregate tumor tissues to harvest TIL to keep the lymphocyte surface intact.

**Figure 2:**
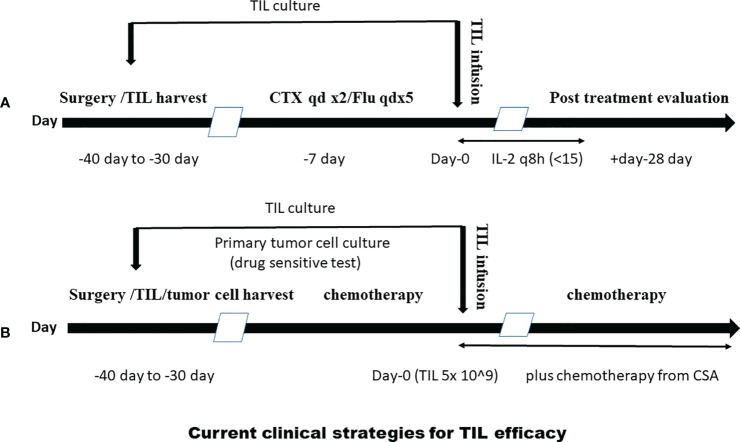
Clinical strategies for TIL efficacy. **(A)** A lympho-depleting regimen adding TIL treatment to increase TIL efficacy. **(B)** A clinical procedure combining TILs with sensitive chemotherapeutic agents, which were screened using chemo-sensitivity assay (CSA) from TIL cytotoxicity experiment of patients’ autogenous tumor cell to increase treatment response.

As mentioned above, we discovered in our early research that TILs have variable efficacy in the treatment of solid tumors: we used some strategies to resolve some questions, as shown in **Figure 1**, in a clinical laboratory, and, as shown in **Figure 2**, in clinical TIL application (93–100).

### Cleaning inhibiting factors

As mentioned above, the results of silencing TILs showed that the supernatant of TILs’ medium and mixed autologous tumor cells contained inhibitory factors. To overcome these inhibitory factors, we investigated some cleaning methods, such as removing autologous tumor cells during TIL culture to obtain a good response to increasing cytotoxicity against autologous tumor cells derived from solid tumors (36). After a long period of research, now clinical scientists and clinical immunologists understand that there are at least two broad categories to keep TIL quiescence – internal factors and external factors, as described above – which are required to block, such as the PD-1 antibody blocker (101–105).

### Increasing immune response to tumor cells

In our early research, to increase immune responses to kill tumor cells, we studied transduced retroviral vectors with TNF-α genes in TILs to increase TIL cytotoxicity function (93–96). To increase immune responses to tumor heterogeneous antigens, some clinical experiments remodeled tumor neo-antigens to T-cells, whereas we studied TIL responses with tumor antigen response with distinct networks (79). For example, after profiling gene expressions by establishing single-cell gene expressions and analyzing molecular expressions of heterogeneous responses of TIL CD8 cells, we found silent gene expressions by TIL CD8 cells such as Tob-1, KLF2, TGFB1, and ERF in solid tumors, which will be discussed below.

### Increasing TIL contact tumor cells

Although adoptive cell therapy using *ex vivo*-activated autologous TIL intravenous infusion is considered one of the promising approaches, early adoptive T-cell immunization is considered to be effective only in some clinical patients with solid tumors. To increase TIL for efficient contact with tumor cells, two studies were performed on TIL-contacted tumor cells. As mentioned above, we have found that early triple enzymes (collagenase IV, hyaluronidase V, and Dnase I) disaggregating solid tumors will influence TIL function; we then modified formulations such as collagenase IV only under mild digestion conditions to keep T-cell intactness from solid tumors (4–7, 106–108). Moreover, my colleagues also used cultured TILs by directly injecting them into a patient’s tumor site to increase TIL-contact tumor cells (14–18). In the analysis of 68 patients with ovarian cancer and other female malignant tumors, intravenous injection of TILs could induce immunity by activating cellular immunity, thereby improving 1-year survival; the anticancer effect of the local injecting group was higher than that of the intravenous group compared with the intravenous and local injection groups of TILs (109, 110).

### Increasing TIL efficacy to tumor cells in clinical application

As mentioned above, TIL treatment of melanoma achieved only a 17% complete remission in an early NCI study, whereas the addition of lymphatic depletion can greatly increase the response ratio (31, 32, 111). Clinically, physicians used a lympho-depleting chemotherapy regimen resulting in a 48% response rate in a melanoma clinical trial from NCI (**Figure 2A**) (33, 112).

To increase TIL efficacy, we have also developed a clinical procedure to combine TILs with sensitive chemotherapeutic agents, which were screened by chemo-sensitivity testing (CST) or chemo-sensitivity assay (CSA) from a patient’s autogenous tumor cell (7, 113), as shown in **Figure 2B**.

## New generation of TIL therapy-personalized immunotherapy

**Figure 3:**
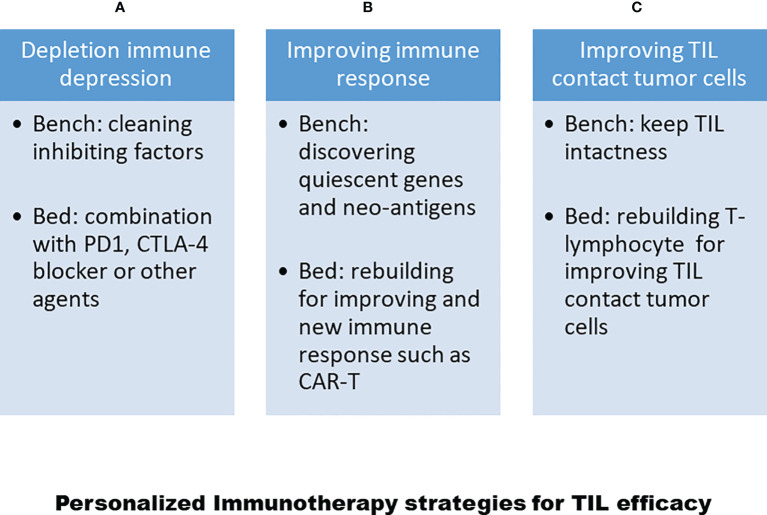
Strategies for personalized TIL therapy to increase TIL efficacy. **(A)** Depleting immune-depression regimen including clean immune depression in experiments and add PD1 or CTLA4 blocker. **(B)** Improving TIL immune response in experiments such as discover quiescent genes for TIL and neo-antigens from tumor cells, and rebuilding immune responses for TILs or setting up Car-T or TCT-T cells. **(C)** Maintaining T-cell intactness and improving T-cell homing into tumor sites.

**Figure 4:**
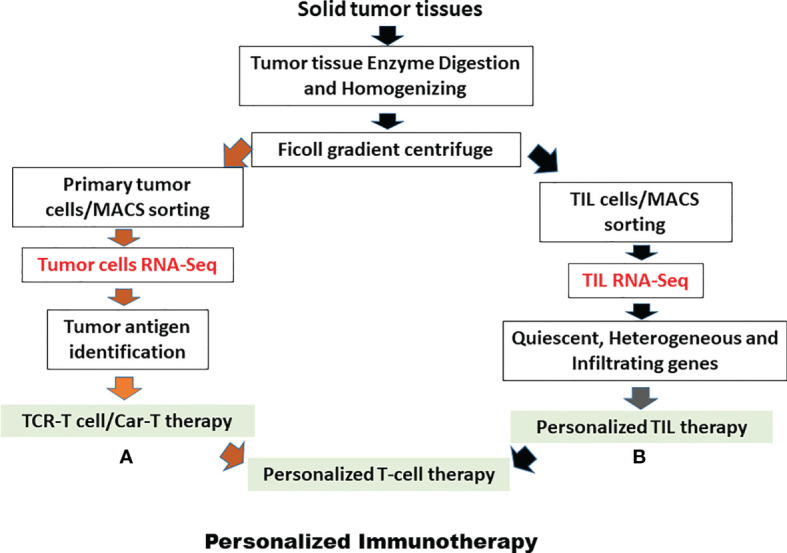
Personalized TIL therapy or personalized T-cell therapy. **(A)** Harvesting tumor cells from solid tumor tissues to run RNA-seq to discover neo-antigen or other related genes. **(B)** Harvesting TILs from solid tumor tissues to run RNA-seq to discover quiescent genes, heterogamous response genes, and infiltrating and homing genes. A pink arrow represents primary tumor cell procedures for performing personalized immunotherapy and a dark arrow represents TIL procedures for performing personalized TIL immunotherapy. Red represents running RNA-seq for both TIL and primary tumor cells.

As noted above, there are at least three main reasons for the variable efficacy of TILs in the treatment of solid tumor diseases. Now, more and more methods and issues of T-lymphocyte treatment will be discovered for the treatment of solid tumors, as shown in **Figure 3**. Moreover, after the development of genomics and proteomics to clinical immunotherapy, more and more targeted drugs with their mechanisms have been discovered for the treatment of tumor diseases. Now is a good time to develop personalized TIL therapies for solid tumor diseases (113). In particular, three achievements, as described above, will lead to personalized TIL R&D. First, single-cell technology has matured, as reported by most clinical laboratories. In addition, we have developed the single-cell techniques for over 15 years from single tumor cells and single TILs from solid tumors, and have identified some genes associated with TIL silencing (114, 115). Second, Dr Rosenberg has identified different SNPs in certain genes in clinical trials from different clinical responses (116). Third, we also used the TIL pathway and artificial intelligence analysis to address the clinical administration of solid cancer (117–119). All in all, following clinical genomics with single-cell technology and artificial intelligence networks, personalized T-cell therapy can be seen in **Figure 4**.

A good immunotherapy should have the characteristics of high efficacy, few side effects and low economic cost for every tumor patient. Based on those considerations, these developments in future immunotherapy require the development of affordable treatments, tolerable side effects and responsible effects for each patient in optimal cell culture and correct clinical treatment. To be sure, individualized T-cells are required for optimal culture in the laboratory and the correct treatment conditions in the clinic. Finally, all efforts in adoptive T-cell immunotherapy focus on establishing optimal T-cell cultures in the laboratory, choosing the correct mode of administration in the clinic, considering affordability, and improving availability to each patient (120, 121).

## Author contributions

The author confirms being the sole contributor of this work and has approved it for publication.

## Funding

Study TIL and primary tumor cell more than 30 years so that the works were funded as different funds such as the National Natural Science Foundation of China(No.39370706), USA IRG-91-022-09 for single cell TIL genomic analysis and tumor cell genomics analysis (PO-1 75606-3) co-work with Dr. Preisler and RO1 CA60086 and so on.

## Acknowledgments

I have studied and set up TIL cultures from solid tumors forclinical application for more than 30 years. All acknowledgements are for my colleagues. Mention of trade names or commercial products in this article is solely for the purpose of providing specific information and does not imply recommendation.

## Conflict of interest

The author declares that the research was conducted in the absence of any commercial or financial relationships that could be construed as a potential conflict of interest.

## Publisher’s note

All claims expressed in this article are solely those of the authors and do not necessarily represent those of their affiliated organizations, or those of the publisher, the editors and the reviewers. Any product that may be evaluated in this article, or claim that may be made by its manufacturer, is not guaranteed or endorsed by the publisher.
